# Social inequalities in multimorbidity patterns in southern Spain: findings from the DEMMOCAD survey

**DOI:** 10.1093/eurpub/ckac130.199

**Published:** 2022-10-25

**Authors:** J Carretero-Bravo, ME Ortega-Martín, B Ramos-Fiol, V Suárez-Lledó, J Álvarez-Gálvez

**Affiliations:** 1 Biomedicine, Biotechnology and Public Health, University of Cadiz, Cadiz, Spain; 2 Clinical Medicine and Public Health, University of Granada, Granada, Spain

## Abstract

**Background:**

Multimorbidity (MM) is associated with lower quality of life, greater disability, and higher use of health services. It is a complex problem that is difficult to capture due to the broad spectrum of concurrent chronic diseases involved. There is a need to identify and characterize patterns of chronic conditions in the local context of specific population groups. The DEMMOCAD project aims to respond to this knowledge gap by detecting patterns of MM and their inequalities in the province of Cadiz (Spain).

**Methods:**

A cross-sectional study was carried out by means of telephone interviews with people over 50 years of age. The final sample was 1592 individuals with MM. A latent class analysis was carried out to identify patterns of MM from 31 chronic conditions. First, the appropriate number of classes was established, considering model fit indices, class size, and clinical interpretability. Subsequently, covariates were introduced into the model using the three-step approach, a technique that minimizes biases in the multinomial regression model.

**Results:**

Preliminary analyses of the goodness-of-fit indices of the model derived five MM patterns (entropy = 0.727): (C1) mild MM; (C2) cardiovascular; (C3) musculoskeletal; (C4) musculoskeletal plus mental; and (C5) complex MM. Compared with class C1, persons in class C5 were significantly older and less educated, class C4 had a lower income, class C3 was smokers and disabled, and class C2 was characteristic among older males. All four classes also showed lower scores on mental and physical dimensions of the SF12 scale compared to class C1.

**Conclusions:**

In addition to providing an adjusted characterization of the population of the area analyzed, these initial findings highlight the existence of social inequalities in multimorbidity at the local level that should be addressed by implementing policies targeting the most vulnerable groups in Cadiz (low socioeconomic status groups, people with disabilities, and the elderly).

**Key messages:**

- Five patterns of multimorbidity were identified in the province of Cadiz (Spain).
- Tailored policies are needed to reduce social inequalities in multimorbidity among vulnerable groups in this local area.

